# Supplementary material for: Modeling glioblastoma heterogeneity as a dynamic network of cell states
Source: Mol Syst Biol. 2021 Sep 16;17(9):e10105. doi: 10.15252/msb.202010105 (PMC8444284; doi:10.15252/msb.202010105)
Supplement: Supplementary file 5 — Source Data for Figure 3 [file MSB-17-e10105-s001.zip › Figure3A_sourcedata/GSEA_3065/hallmarks_state2.GseaPreranked.1623416269049/HALLMARK_FATTY_ACID_METABOLISM.html]

Details for gene set HALLMARK\_FATTY\_ACID\_METABOLISM[GSEA]

|  || Dataset | state2 |
| Phenotype | NoPhenotypeAvailable |
| Upregulated in class | na\_pos |
| GeneSet | HALLMARK\_FATTY\_ACID\_METABOLISM |
| Enrichment Score (ES) | 0.36820868 |
| Normalized Enrichment Score (NES) | 1.2382838 |
| Nominal p-value | 0.13216957 |
| FDR q-value | 0.19766736 |
| FWER p-Value | 0.994 |
Table: GSEA Results Summary

  

Fig 1: Enrichment plot: HALLMARK\_FATTY\_ACID\_METABOLISM      
 Profile of the Running ES Score & Positions of GeneSet Members on the Rank Ordered List

  

| PROBE | GENE SYMBOL | GENE\_TITLE | RANK IN GENE LIST | RANK METRIC SCORE | RUNNING ES | CORE ENRICHMENT || 1 | ACAT2 |  |  | 180 | 0.294 | 0.0217 | Yes |
| 2 | YWHAH |  |  | 221 | 0.271 | 0.0552 | Yes |
| 3 | PTS |  |  | 359 | 0.215 | 0.0705 | Yes |
| 4 | LDHA |  |  | 506 | 0.179 | 0.0799 | Yes |
| 5 | HSP90AA1 |  |  | 600 | 0.164 | 0.0928 | Yes |
| 6 | ECI1 |  |  | 607 | 0.162 | 0.1148 | Yes |
| 7 | MDH2 |  |  | 615 | 0.161 | 0.1365 | Yes |
| 8 | CBR3 |  |  | 616 | 0.161 | 0.1589 | Yes |
| 9 | ACADM |  |  | 715 | 0.148 | 0.1691 | Yes |
| 10 | HADH |  |  | 778 | 0.141 | 0.1822 | Yes |
| 11 | KMT5A |  |  | 821 | 0.136 | 0.1967 | Yes |
| 12 | SMS |  |  | 846 | 0.133 | 0.2127 | Yes |
| 13 | IDI1 |  |  | 878 | 0.130 | 0.2275 | Yes |
| 14 | NCAPH2 |  |  | 910 | 0.127 | 0.2419 | Yes |
| 15 | SUCLG1 |  |  | 1076 | 0.113 | 0.2399 | Yes |
| 16 | HSD17B10 |  |  | 1143 | 0.109 | 0.2480 | Yes |
| 17 | FH |  |  | 1155 | 0.108 | 0.2619 | Yes |
| 18 | ECHS1 |  |  | 1322 | 0.097 | 0.2576 | Yes |
| 19 | HSPH1 |  |  | 1399 | 0.092 | 0.2623 | Yes |
| 20 | ECI2 |  |  | 1422 | 0.091 | 0.2727 | Yes |
| 21 | BLVRA |  |  | 1465 | 0.088 | 0.2805 | Yes |
| 22 | GRHPR |  |  | 1610 | 0.081 | 0.2764 | Yes |
| 23 | IDH3B |  |  | 1635 | 0.080 | 0.2851 | Yes |
| 24 | NBN |  |  | 1657 | 0.079 | 0.2938 | Yes |
| 25 | NTHL1 |  |  | 1730 | 0.076 | 0.2967 | Yes |
| 26 | PCBD1 |  |  | 1732 | 0.076 | 0.3072 | Yes |
| 27 | CBR1 |  |  | 1744 | 0.075 | 0.3164 | Yes |
| 28 | PDHB |  |  | 1825 | 0.071 | 0.3178 | Yes |
| 29 | MDH1 |  |  | 1845 | 0.070 | 0.3256 | Yes |
| 30 | ODC1 |  |  | 1913 | 0.068 | 0.3279 | Yes |
| 31 | ACAA2 |  |  | 1924 | 0.067 | 0.3362 | Yes |
| 32 | ALDH9A1 |  |  | 1951 | 0.066 | 0.3427 | Yes |
| 33 | SDHD |  |  | 1999 | 0.065 | 0.3467 | Yes |
| 34 | GPD2 |  |  | 2101 | 0.061 | 0.3444 | Yes |
| 35 | CA2 |  |  | 2111 | 0.061 | 0.3519 | Yes |
| 36 | GCDH |  |  | 2137 | 0.060 | 0.3576 | Yes |
| 37 | APEX1 |  |  | 2239 | 0.056 | 0.3545 | Yes |
| 38 | MGLL |  |  | 2293 | 0.054 | 0.3564 | Yes |
| 39 | CCDC58 |  |  | 2371 | 0.051 | 0.3553 | Yes |
| 40 | ADSL |  |  | 2425 | 0.050 | 0.3566 | Yes |
| 41 | RAP1GDS1 |  |  | 2476 | 0.048 | 0.3580 | Yes |
| 42 | MIF |  |  | 2504 | 0.047 | 0.3618 | Yes |
| 43 | S100A10 |  |  | 2608 | 0.044 | 0.3569 | Yes |
| 44 | HSDL2 |  |  | 2644 | 0.043 | 0.3592 | Yes |
| 45 | DECR1 |  |  | 2696 | 0.042 | 0.3595 | Yes |
| 46 | HSD17B4 |  |  | 2829 | 0.038 | 0.3507 | Yes |
| 47 | CRAT |  |  | 2856 | 0.038 | 0.3532 | Yes |
| 48 | DLD |  |  | 2865 | 0.037 | 0.3575 | Yes |
| 49 | UGDH |  |  | 2897 | 0.036 | 0.3593 | Yes |
| 50 | UBE2L6 |  |  | 2901 | 0.036 | 0.3640 | Yes |
| 51 | PDHA1 |  |  | 2916 | 0.036 | 0.3676 | Yes |
| 52 | PSME1 |  |  | 2957 | 0.035 | 0.3681 | Yes |
| 53 | ENO2 |  |  | 3006 | 0.034 | 0.3677 | Yes |
| 54 | SUCLG2 |  |  | 3045 | 0.033 | 0.3682 | Yes |
| 55 | AADAT |  |  | 3105 | 0.032 | 0.3663 | No |
| 56 | SDHA |  |  | 3219 | 0.029 | 0.3583 | No |
| 57 | ADIPOR2 |  |  | 3549 | 0.023 | 0.3262 | No |
| 58 | HCCS |  |  | 3622 | 0.022 | 0.3215 | No |
| 59 | PRDX6 |  |  | 3639 | 0.022 | 0.3228 | No |
| 60 | METAP1 |  |  | 3660 | 0.021 | 0.3236 | No |
| 61 | MCEE |  |  | 3893 | 0.017 | 0.3012 | No |
| 62 | UROS |  |  | 3995 | 0.015 | 0.2925 | No |
| 63 | ECH1 |  |  | 4011 | 0.015 | 0.2930 | No |
| 64 | BPHL |  |  | 4023 | 0.015 | 0.2940 | No |
| 65 | GSTZ1 |  |  | 4096 | 0.014 | 0.2882 | No |
| 66 | IDH3G |  |  | 4210 | 0.012 | 0.2778 | No |
| 67 | HMGCS1 |  |  | 4272 | 0.011 | 0.2727 | No |
| 68 | NSDHL |  |  | 4406 | 0.009 | 0.2597 | No |
| 69 | ALDOA |  |  | 4435 | 0.008 | 0.2579 | No |
| 70 | HMGCL |  |  | 4735 | 0.004 | 0.2263 | No |
| 71 | HADHB |  |  | 4810 | 0.003 | 0.2188 | No |
| 72 | SUCLA2 |  |  | 4860 | 0.002 | 0.2137 | No |
| 73 | DLST |  |  | 4897 | 0.001 | 0.2100 | No |
| 74 | HSD17B7 |  |  | 5217 | -0.004 | 0.1764 | No |
| 75 | BCKDHB |  |  | 5263 | -0.004 | 0.1721 | No |
| 76 | SDHC |  |  | 5266 | -0.004 | 0.1725 | No |
| 77 | HSD17B11 |  |  | 5658 | -0.011 | 0.1322 | No |
| 78 | UROD |  |  | 5835 | -0.014 | 0.1152 | No |
| 79 | ALAD |  |  | 5853 | -0.014 | 0.1154 | No |
| 80 | CPOX |  |  | 5870 | -0.014 | 0.1156 | No |
| 81 | CPT2 |  |  | 5964 | -0.016 | 0.1079 | No |
| 82 | ACO2 |  |  | 6010 | -0.017 | 0.1054 | No |
| 83 | FASN |  |  | 6119 | -0.019 | 0.0964 | No |
| 84 | ACAA1 |  |  | 6129 | -0.019 | 0.0981 | No |
| 85 | IDH1 |  |  | 6208 | -0.020 | 0.0925 | No |
| 86 | MLYCD |  |  | 6396 | -0.023 | 0.0758 | No |
| 87 | ACOT8 |  |  | 6528 | -0.026 | 0.0653 | No |
| 88 | EPHX1 |  |  | 6547 | -0.026 | 0.0670 | No |
| 89 | G0S2 |  |  | 6695 | -0.029 | 0.0554 | No |
| 90 | ETFDH |  |  | 6820 | -0.032 | 0.0466 | No |
| 91 | ELOVL5 |  |  | 7020 | -0.037 | 0.0304 | No |
| 92 | D2HGDH |  |  | 7056 | -0.038 | 0.0320 | No |
| 93 | DHCR24 |  |  | 7171 | -0.041 | 0.0255 | No |
| 94 | ACSL1 |  |  | 7456 | -0.049 | 0.0019 | No |
| 95 | PPARA |  |  | 7537 | -0.051 | 0.0005 | No |
| 96 | ACADVL |  |  | 7628 | -0.054 | -0.0016 | No |
| 97 | GABARAPL1 |  |  | 7630 | -0.054 | 0.0059 | No |
| 98 | ALDH3A2 |  |  | 7763 | -0.059 | -0.0001 | No |
| 99 | ACSL4 |  |  | 7960 | -0.065 | -0.0120 | No |
| 100 | HIBCH |  |  | 8030 | -0.068 | -0.0098 | No |
| 101 | GLUL |  |  | 8307 | -0.083 | -0.0278 | No |
| 102 | OSTC |  |  | 8367 | -0.087 | -0.0220 | No |
| 103 | ACOX1 |  |  | 8525 | -0.098 | -0.0251 | No |
| 104 | LGALS1 |  |  | 8680 | -0.110 | -0.0263 | No |
| 105 | RDH11 |  |  | 9108 | -0.167 | -0.0487 | No |
| 106 | PTPRG |  |  | 9127 | -0.172 | -0.0266 | No |
| 107 | ERP29 |  |  | 9212 | -0.197 | -0.0081 | No |
| 108 | SERINC1 |  |  | 9303 | -0.233 | 0.0147 | No |
Table: GSEA details [plain text format]

  

Fig 2: HALLMARK\_FATTY\_ACID\_METABOLISM: Random ES distribution      
 Gene set null distribution of ES for **HALLMARK\_FATTY\_ACID\_METABOLISM**
